# Dr. John Little Moffatt

**Published:** 1917-04

**Authors:** 


**Dr. John Little Moffatt,** N. Y. Homoeopathic 1877, died at his home in Ithaca, Feb. 18, of tuberculosis, aged 63. He was a laryngologist, having been editor of Ophthalmology, Otology and Laryngology from 1901 to 1904, and of the Homoeopathic Eye, Ear and Throat Journal 1905-1910.

